# Rhythm vs. rate control for treatment of postoperative atrial fibrillation after cardiac surgery: a systematic review and meta-analysis of randomized controlled trials

**DOI:** 10.3389/fcvm.2026.1820175

**Published:** 2026-07-14

**Authors:** Mohammad S. Dairi, Mohammed Tarabzoni, Christopher Tarola, Herman Sehmbi, Hassan Alwafi, Saeed M. Alghamdi, Sariya Khan, Waleed Talal Alotaibi, Ahmed F. Hegazy

**Affiliations:** 1 Department of Medicine, College of Medicine, Umm Al-Qura University, Makkah, Saudi Arabia; 2 Cardiovascular and Thoracic Surgery Department, King Salman Heart Centre, King Fahad Medical City (KFMC), Riyadh, Saudi Arabia; 3 Department of Surgery, Sunnybrook Health Sciences Centre, University of Toronto, Toronto, ON, Canada; 4 Department of Anesthesia, Waterloo Regional Health Network, McMaster University, Hamilton, ON, Canada; 5 Department of Pharmacology and Toxicology, College of Medicine, Umm Al-Qura University, Makkah, Saudi Arabia; 6 Clinical Technology Department, Respiratory Care Program, Faculty of Applied Medical Sciences, Umm Al-Qura University, Makkah, Saudi Arabia; 7 General Medicine Practice Program, Batterjee Medical College, Jeddah, Saudi Arabia; 8 Department of Medicine, College of Medicine, Umm Al-Qura University, Makkah, Saudi Arabia; 9 Division of Critical Care Medicine, Department of Medicine, University of Western Ontario, London, Ontario, ON, Canada; 10 Cardiac Surgery Intensive Care Unit, Department of Critical Care Medicine, Madinah Cardiac Centre, Madinah, Saudi Arabia

**Keywords:** cardiac surgery, meta-analysis, new-onset atrial fibrillation, postoperative atrial fibrillation, rate control, rhythm control, systematic review

## Abstract

**Background:**

We aimed to compare the clinical impact of treatment with a rhythm control strategy to a rate control strategy in postcardiac surgery atrial fibrillation patients.

**Methods:**

A comprehensive search of MEDLINE, Embase, Cochrane Central Register of Controlled Trials, CINAHL, Web of Science, Scopus, ProQuest Dissertations, and ClinicalTrials.gov was conducted from inception to October 2025. Our meta-analysis included randomized controlled trials (RCTs) comparing therapeutic rhythm control interventions with rate control interventions. We used the Cochrane risk-of-bias tool to appraise the quality of included RCTs, the GRADE framework to evaluate the strength of the evidence, and adhered to the PRISMA guidelines for reporting.

**Results:**

Eight RCTs (*n* = 894 patients) met the inclusion criteria. There was no difference in hospital length of stay (4 RCTs) between rhythm control and rate control [MD: −0.41 days (95% CI: −3.23, 2.42)]. An aggressive rhythm control strategy (ibutilide, procainamide, propafenone, or electric cardioversion) was associated with higher odds of in-hospital conversion to sinus rhythm [OR: 4.01 (95% CI: 1.30, 12.39)] and a higher risk of medication-related adverse events (hypotension, bradycardia, and syncope) [RR: 3.05 (95% CI: 1.05, 8.89)].

**Conclusion:**

Among postcardiac surgery patients with new-onset atrial fibrillation, there was no evidence that a rhythm control treatment strategy resulted in better outcomes than a rate control strategy.

**Systematic Review Registration:**

https://www.crd.york.ac.uk/PROSPERO/view/CRD42019128559, identifier CRD42019128559.

## Introduction

Postoperative atrial fibrillation (POAF) is a common complication after cardiac surgery that can lead to significant morbidity and mortality (1). Its incidence is highest after combined valve surgery with coronary artery bypass grafting (CABG) (50%), followed by valve surgery in isolation (30%–40%), followed by CABG in isolation (15%–25%) (2). Patient characteristics that increase the likelihood of developing new-onset POAF include advanced age, reduced left ventricular systolic function, male gender, pre-existing hypertension, and renal dysfunction (3). POAF after cardiac surgery is currently thought to be driven by multiple factors: the inflammatory response of surgery, neurohormonal activation, and the presence of a structural or metabolic substrate for its development (4).

Despite its largely self-limiting course, new-onset POAF after cardiac surgery is an independent predictor of serious adverse outcomes, both short-term and long-term. Short-term adverse outcomes include hemodynamic instability, acute congestive heart failure (CHF), pulmonary edema, and increased hospital length of stay (LOS). Long-term adverse outcomes include increased risk of stroke and mortality (5, 6).

Treatment strategies for POAF include a rhythm-control approach, focusing on conversion to normal sinus rhythm (NSR), or a rate-control approach. A rhythm-control strategy may hasten in-hospital reversion to NSR, reduce the incidence of persistent atrial fibrillation, and obviate the need for anticoagulation. Conversely, a rate-control approach may help avoid side effects and drug interactions associated with rhythm-control medications and protect against rapid ventricular rates until spontaneous reversion to NSR occurs (5, 7).

A previously published systematic review comparing rhythm vs. rate control in patients with POAF after cardiac surgery identified important gaps in the literature. Our systematic review and meta-analysis aims to address these gaps. The previous review noted a paucity of randomized trials, heterogeneity in interventions, variability in follow-up duration, and a lack of evaluation of long-term clinical outcomes beyond the early postoperative period (8). Our study employed a more comprehensive search strategy, expanded the scope of relevant outcomes, and conducted additional analyses not undertaken in the previous study.

## Methods

The protocol for this systematic review and meta-analysis was registered and is publicly available in the International Prospective Register of Systematic Reviews (PROSPERO) (CRD42019128559). We reported all findings in accordance with the Preferred Reporting Items for Systematic Reviews and Meta-Analyses (PRISMA) guidelines statement (9).

### Outcomes

**Figure 1:**
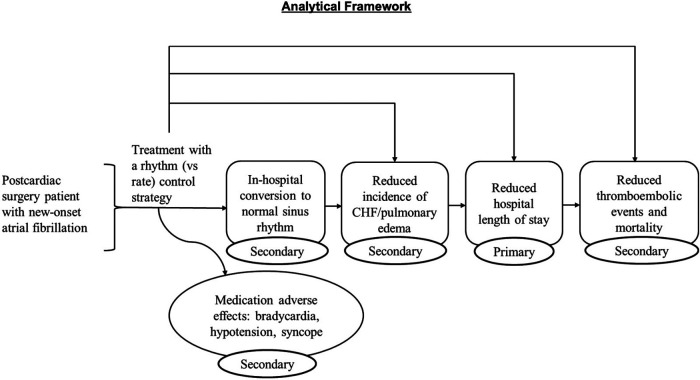
Analytical framework examining the impact of a rhythm control strategy compared to a rate control strategy on various outcome variable.

We examined the impact of implementing a therapeutic rhythm control strategy vs. a rate control strategy in postcardiac surgery patients with new-onset atrial fibrillation. Postoperative atrial fibrillation in this population has been shown to prolong hospital length of stay (LOS). We therefore compared the impact of each treatment strategy on hospital LOS (primary outcome). Secondary outcomes included the odds of in-hospital conversion to normal sinus rhythm (NSR) and the risk of medication-related hemodynamic side effects (hypotension, bradycardia, and syncope). Given the negative inotropic effects of rhythm and rate control medications, we also examined the effect of these two strategies on the risk of developing congestive heart failure (CHF) or pulmonary edema during the index operative admission (Table 1—Outcome Definitions). Long-term secondary outcomes included the odds of thromboembolic events and death during the study follow-up period (Figure 1—Analytical Framework).

**Table 1:** Outcome definitions for systematic review and meta-analaysis.

Outcome	Definition	Measurement unit
Hospital length of stay	Postoperative or postrandomization hospital length of stay for the index cardiac surgery admission	Days
In-hospital conversion to normal sinus rhythm	Number of patients converted to normal sinus rhythm at latest reported time-point during index cardiac surgery admission	Frequency
Medication adverse effects	Number of patients developing any of the following: hypotension, bradycardia, or syncope during study follow-up	Frequency
Incidence of congestive heart failure & pulmonary edema	Number of patients developing new-onset heart failure or pulmonary edema during study follow-up	Frequency
Thromboembolic events	Number of patients developing any thromboembolic event (cerebrovascular or non-cerebrovascular) during study follow-up	Frequency
Mortality	Number of postcardiac surgery deaths during study follow-up	Frequency

### Search strategy

We searched Medline & Medline in-process (Ovid), PubMed-NLM, Embase (Ovid), CENTRAL, Cumulative Index of Nursing and Allied Health Literature (CINAHL), Web of Science, Scopus, ProQuest Dissertations and Theses Global from inception to October 2025 using a pre-defined search strategy (Supplementary Appendix 2). We also searched the clinical trial registry (www.ClinicalTrials.gov) for ongoing and completed but unpublished studies. This search was conducted by a professional research librarian using both controlled vocabulary and sensitive keyword search terms. Related subject headings (controlled vocabulary) for each database were also identified and included in the search. We did not impose any language restrictions on the search.

### Study selection

We sought full-text published RCT manuscripts using a predefined Population/Intervention/Comparator/Outcome (PICO) framework. Our population was adult patients (≥18 years) with POAF after cardiac surgery. The intervention of interest was rhythm control, defined as attempts to convert to normal sinus rhythm by any method, whether pharmacological (e.g., amiodarone, propafenone, ibutilide, sotalol) or by synchronized electrical conversion. Our comparator was rate-control interventions, defined as pharmacological strategies aimed at reducing heart rate using beta-blockers, calcium channel blockers, or digoxin. The primary outcome was postoperative or post-randomization hospital LOS in days. Secondary outcomes included in-hospital rates of conversion to NSR at the latest reported time-point, antiarrhythmic medication adverse effects (specifically hypotension, bradycardia, and syncope), rates of thrombo-embolic events (cerebrovascular and non-cerebrovascular), CHF/pulmonary edema, and all-cause mortality during the follow-up period. We excluded studies comparing two rate-control strategies or two rhythm-control strategies, case series, case reports, observational studies, studies examining prophylactic interventions, and pediatric surgery studies.

### Study screening

All articles were screened independently by two reviewers (MD & MT). Any discrepancies at the screening stage were resolved under the supervision of a third reviewer (AH) through discussion and consensus.

### Data extraction and quality assessment

Two reviewers (MD and CT) independently extracted data from eligible RCTs using predefined data extraction forms. For missing study-level data, we corresponded with three primary investigators. Two authors did not respond, while the third replied that he no longer had access to study data due to his relocation. When feasible, patient-level numerical data was extracted from plots using a web-based data extraction tool, WebPlotDigitizer (Ankit Rohatgi, WebPlotDigitizer Version 4.2, San Francisco, California, USA), which is known for high intercoder reliability and validity (10). The accuracy of the extracted data was then verified by a third reviewer (AH).

Two reviewers (MD and MT) independently assessed the risk of bias of the included studies using the Revised Cochrane Risk of Bias Tool for Randomized Trials 2 (RoB 2). Any disagreements were resolved through discussion and adjudication by a third reviewer (AH) (9). The certainty of evidence for each outcome was assessed using the Grading of Recommendations, Assessment, Development and Evaluations (GRADE) approach. This framework evaluates the quality of evidence based on study limitations, inconsistency, indirectness, imprecision, and publication bias. The overall certainty of evidence was categorized as high, moderate, low, or very low.

### Statistical analysis

Dichotomous outcomes were extracted as events/non-events, and continuous outcomes were extracted as means and standard deviations. When only medians and interquartile ranges were available, the methods described by Wan et al. were used to estimate means and standard deviations (11). Anticipating numerous sources of clinical heterogeneity, including diverse cardiac surgical procedures, the type and dosing of pharmacological agents, and patient demographics, we used a DerSimonian & Laird random-effects model to pool effect-size data (12). Effect summaries were presented as Forest plots, and statistical heterogeneity was assessed using Cochran's *Q* test and *I*^2^. Heterogeneity was considered “high” when Cochran's *Q* test yielded a statistically significant *p*-value (*p* ≤ 0.05) or when I^2^ was > 75% (13). When substantial heterogeneity was observed, subgroup analyses were conducted to further explore potential causes. Publication bias was assessed by examining funnel plot asymmetry using Egger's regression test (14). The robustness of GRADE assessments was tested using sensitivity (leave-one-out) analyses. Statistical analyses were performed using Review Manager (RevMan) Version 5.4 (Copenhagen: The Nordic Cochrane Centre, The Cochrane Collaboration, 2020) and R version 4.0.0 (R Foundation for Statistical Computing, Vienna, Austria) with the Metafor package for meta-analysis.

## Results

### Study characteristics

**Figure 2:**
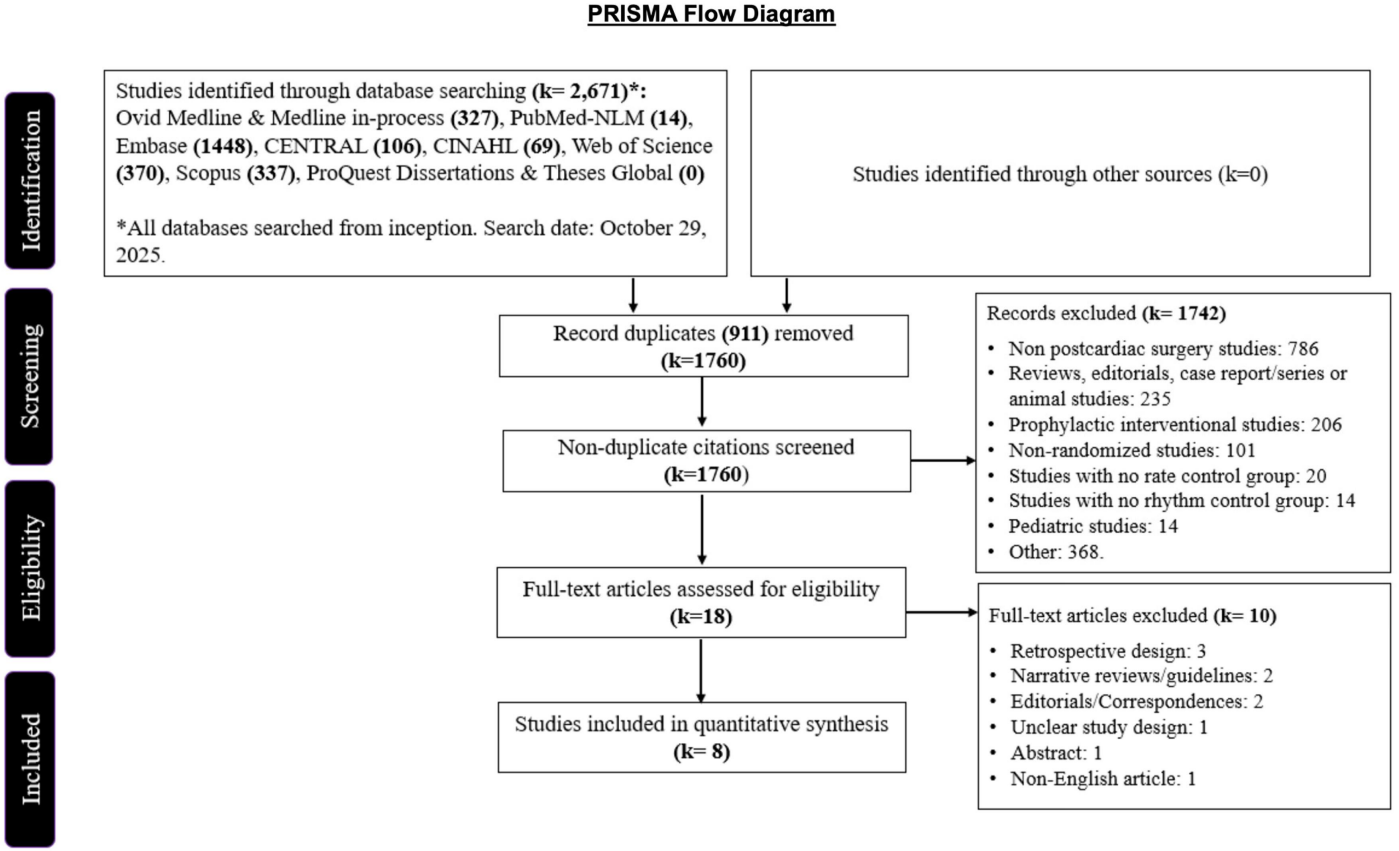
PRISMA flow diagram outlining study identification, screening, eligibility, and inclusion.

A total of 2,671 records were identified through database searches. Study screening and selection proceeded through multiple phases, as shown in the PRISMA flow diagram (Figure 2). After removing duplicates and excluding ineligible articles, eight RCTs (*n* = 894 patients) met the inclusion criteria. Most studies had small-to-medium sample sizes (29–150 patients), with only one study enrolling 523 patients. Rhythm control interventions included using any of sotalol, amiodarone, procainamide, propafenone, ibutilide, flecainide, and synchronized electric cardioversion. Rate control interventions included metoprolol, digoxin, diltiazem, and verapamil. All studies reported the incidence of conversion to normal sinus rhythm and medication-related hypotension. Follow-up durations ranged from 12 h to 60 days. Only four studies reported hospital LOS. Characteristics of the included studies are shown in Table 2.

**Table 2:** Characteristics of included studies.

Study details	Study design	Location	Inclusion criteria and exclusion Criteria	Group	Intervention	Number enrolled	Male/Female	Age mean (SD)	Follow-up duration	Study findings
Campbell et al. 1985	Single-center randomized trial	Australia	Inclusion criteria Post-cardiac surgery Atrial Arrhythmias and Ventricular response >120 Exclusion Criteria Glaucoma or asthma history Severe renal impairment Preoperative arrhythmia or AV block (2nd or 3rd degree) Recent (within the last 48 h) rhythm or rate control medications	Rhythm control	IV Sotalol	20	M: 19, F:1	60.5 (9.1)	12 h	A sealed envelope system concealed allocation, but investigators were not blinded. Sotalol was more effective in conversion to NSR, but digoxin had less hemodynamic adverse effects.
				Rate control	IV Digoxin (IV disopyramide was used only when digoxin had failed)	20	M:15, F:5	63.5 (5.2)		
Cochrane et al. 1994	Single-center randomized trial	Australia	Inclusion criteria Open heart surgery patients with new onset AF that persisted >20 min, with SBP ≥ 85 mmHg and no inotropic support Exclusion Criteria AF prior to surgery Poor LV function on LV ventriculogram (Grade 4) Postoperative beta-blocker administration	Rhythm control	IV Amiodarone (If reversion to NSR had not occurred, then digoxin was added)	15	M:11, F:4	60.2 (no SD)	24 h	Lack of allocation concealment given that randomization was based on hospital record number. Authors did not provide information on blinding. Amiodarone showed similar efficacy to digoxin in conversion to NSR. None of the patients experienced major drug-related adverse events.
				Rate control	IV Digoxin (If reversion to NSR had not occurred, then amiodarone was added)	15	M:10, F:5	65.8 (no SD)		
Gillinov et al. 2016	Multicenter randomized trial	US and Canada (23 Centres)	Inclusion criteria Adult with hemodynamically stable new-onset POAF (after elective surgeries) that persisted for more than 60 min or recurrent episodes of atrial fibrillation during the index hospitalization (≤7 days after surgery) Exclusion Criteria Patients with a prior history of atrial fibrillation were excluded to avoid making changes to their established preoperative medication regimen for atrial fibrillation and anticoagulation.	Rhythm control	Amiodarone (with or without rate control agent. For persistent AF > 24–8 h, DC cardioversion was recommended)	261	M:199, F:62	68.4 (8.4)	60 days	Trial was pre-registered, and the protocol was adhered to (NCT02132767) Block randomization No allocation concealment Open-label trial (not blinded) Adequate duration of follow-up (30 and 60 days) There were no significant differences in the outcomes of interest, including length of hospital stay, complications rate, rate of conversion to NSR, or medication adverse events between both groups
				Rate control	Rate control agents (BB or CCB), with a goal of achieving a resting heart rate of less than 100 bpm. Switching to rhythm control was allowed for hemodynamic or symptom control.	262	M:197, F:65	69.2 (9.8)		
Hejmls et al. 1992	Single-center randomized trial	Denmark	Inclusion criteria AF after open heart surgery Exclusion Criteria Heart block but not bundle branch block Patients receiving digoxin treatment at the time when postoperative atrial fibrillation occurred	Rhythm control	IV Procainamide followed by oral maintenance	15	M:13, F:2	Median: 65 (IQR: 46–74)	12 h	No information provided on blinding or allocation concealment Unclear on the duration of follow-up Procainamide was more effective than digoxin for conversion to normal sinus rhythm and Procainamide group had more drop in systolic blood pressure but with no major clinical consequences
				Rate control	IV Digoxin followed by oral maintenance	15	M:11, F:4	Median: 60 (IQR: 17–72)		
Kamali et al. 2017	Single-center randomized trial	Iran	Inclusion criteria CABG patients (Age 45–80 years old) with no prior history of arrhythmia or on antiarrhythmic medications Exclusion Criteria Patients with atrial fibrillation following CABG and “not responding to treatment” Patients requiring DC shock to treat arrhythmia Patients requiring a procedure other than CABG Patients with a prior history of arrhythmia or using antiarrhythmic medications Age < 45 or >80 years old Emergency CABG patients	Rhythm control	IV Amiodarone	75	M:42, F:33	—	24 h	Double-blinded with allocation concealment (according to the table of randomized numbers) No table provided to compare demographics Metoprolol was more efficacious in achieving conversion to NSR compared to amiodarone However, the metoprolol group had a slightly higher rate of adverse events and longer CSICU LOS
				Rate control	Metoprolol	75	M:37, F:38	—		
Lee et al. 2000	Single-center randomized trial	Canada	Inclusion criteria 18 years and older who had atrial fibrillation for at least 1 h and had no history of paroxysmal atrial fibrillation Exclusion Criteria Received antiarrhythmic therapy within 5 half-lives of the time of random assignment *β*-blockers withdrawn after surgery Patients in cardiogenic shock Creatinine level >200 μg/mmol Serum AST or ALT 4 times the upper limit of normal Conduction abnormalities before randomization Contraindications to anticoagulation	Rhythm control	Sotalol, propafenone, or procainamide, with or without electric cardioversion	27	M:21, F:6	67 (7)	60 days	No information was provided regarding allocation concealment or blinding No overall significant difference in time to conversion to NSR, though the antiarrhythmic group showed faster restoration of NSR. Hospital LOS was shorter in the rhythm arm compared to the rate control arm. Both groups had similar rates of relapse. Certain outcome measures, including CHF and mortality, were not reported in the rate arm
				Rate control	IV diltiazem, BB or digoxin	23	M:18, F:5	70 (5)		
Soucier et al. 2003	Randomized trial	USA (2 Centres)	Inclusion criteria Hemodynamically stable new-onset AF patients hospitalized on the telemetry step-down units after open-heart surgery. AF duration was between 3 and 72 h. Exclusion Criteria Chronic AF prior to surgery Hemodynamically unstable AF AF with a slow ventricular response Patients with an underlying BBB, high-grade AV block, tachycardia/bradycardia syndrome without a functioning pacemaker. Concurrent illness, including untreated overt CHF, pneumonia, hyperthyroidism, hepatitis postoperative angina, clinical evidence of digitalis toxicity. Evidence of myocardial infarction within 7 days of randomization. Patients with untreated hypokalemia (K < 4.0 mEq/L) or hypomagnesemia (Mg < 1.3 mEq/L). Recent (within 5 half-lives) exposure to a type I or III antiarrhythmic drug. Prolonged QTc. A history of Torsades de Pointes. Failure of the patient or the attending physician to consent to the procedure.	Rhythm control	IV ibutilide oral propafenone	30	M:24, F:6	Ibutilide: 76 (6), Propafenone: 70 (9)	14 days	Open label trial No information on allocation concealment Imbalanced group number (more patients in the rhythm control arm compared to the rate control arm) Ibutilide reduced the duration of AF more effectively compared to either propafenone or rate control agents at 24 h, though the recurrence rate was similar No significant differences in the rate of complications or VTE among the groups
				Rate control	Digoxin, beta-blockers and/or calcium channel-blocking agents	12	M:9, F:3	76 (7)		
Wafaa et al. 1989	Single-center randomized trial	UK	Inclusion criteria 18–80 years old patients who had CAGB complicated by atrial tachyarrhythmia (AF, A. Flutter, and AT) within 96 h post-op and lasting >15 min with ventricular response >120 bpm Exclusion Criteria Preoperative atrial tachyarrhythmia 2nd or 3rd degree atrioventricular(AV) block, history of bifasicular block, bundle branch block with any degree of AV block Impaired LV function Treatment with other antiarrhythmics during anesthesia or upon return to ICU Treatment with digoxin or beta-blockers in the 24 h before entering the study Serious renal or liver dysfunction Receiving any investigational drug during the 4-week period before entering the study or antiarrhythmic agents within 3 elimination half-lives of the date of inclusion for the study	Rhythm control	Flecanide IV bolus followed infusion for 24 h. Verapamil 10 mg was given if, after 45 min, the patient failed to revert to SR with VR <100	15	M:15, F:0	61 (8)	24 h	Allocation concealment by opening a numbered sealed envelope) Minor heterogeneity in gender between both group Flecanide was more effective in achieving conversion to NSR and controlling the arrhythmia compared to digoxin Though medication adverse events were more in the flecainide arm, they were minor and responded well to cessation of medication
				Rate control	Digoxin IV 0.5. Verapamil 10 mg was given if, after 45 min, the patient failed to revert to SR with VR <100	14	M: 11, F:3	66 (5)		

### Risk of bias assessment

**Figure 3:**
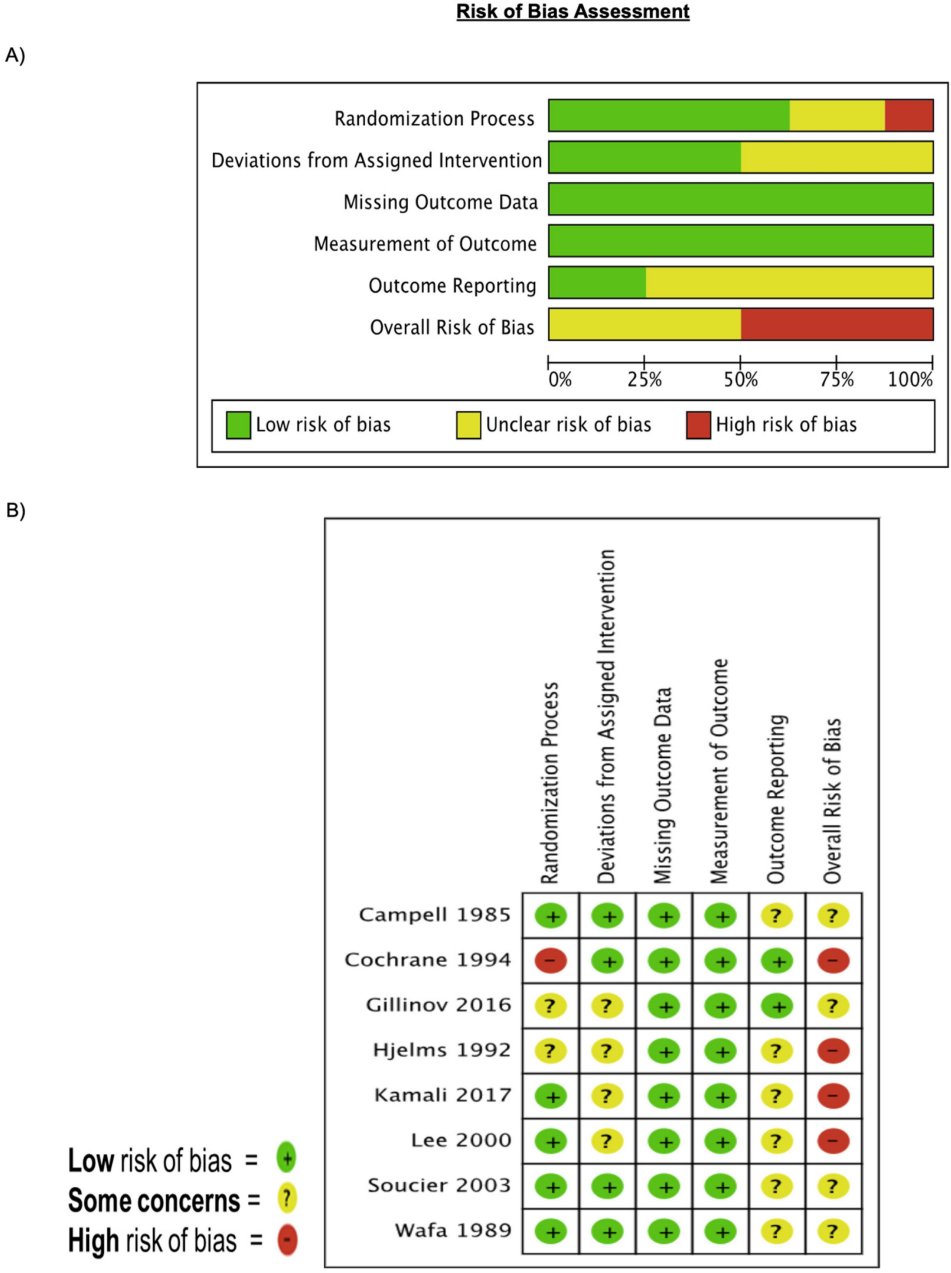
Risk of bias graph and risk of bias individual assessment. **(A)** Cochrane risk of bias graph. **(B)** Summary of risk of bias assessment for individual studies.

Most studies adequately described the methods used for randomization and allocation concealment, except for the study by Cochrane et al. (15). “Some concerns” were identified with blinding of interventions in 4 RCTs (16–19). All studies were judged to be at low risk of missing outcome data or ascertainment bias. Six RCTs, however, were considered at “some risk” of bias from selective outcome reporting (17–22). In summary, 4 RCTs were deemed at an overall “high risk” of bias (15, 17–19), while the remaining four had “some concerns” (16, 20–22) (Figure 3).

### Outcomes

#### Postoperative or post-randomization hospital length of stay

**Figure 4:**
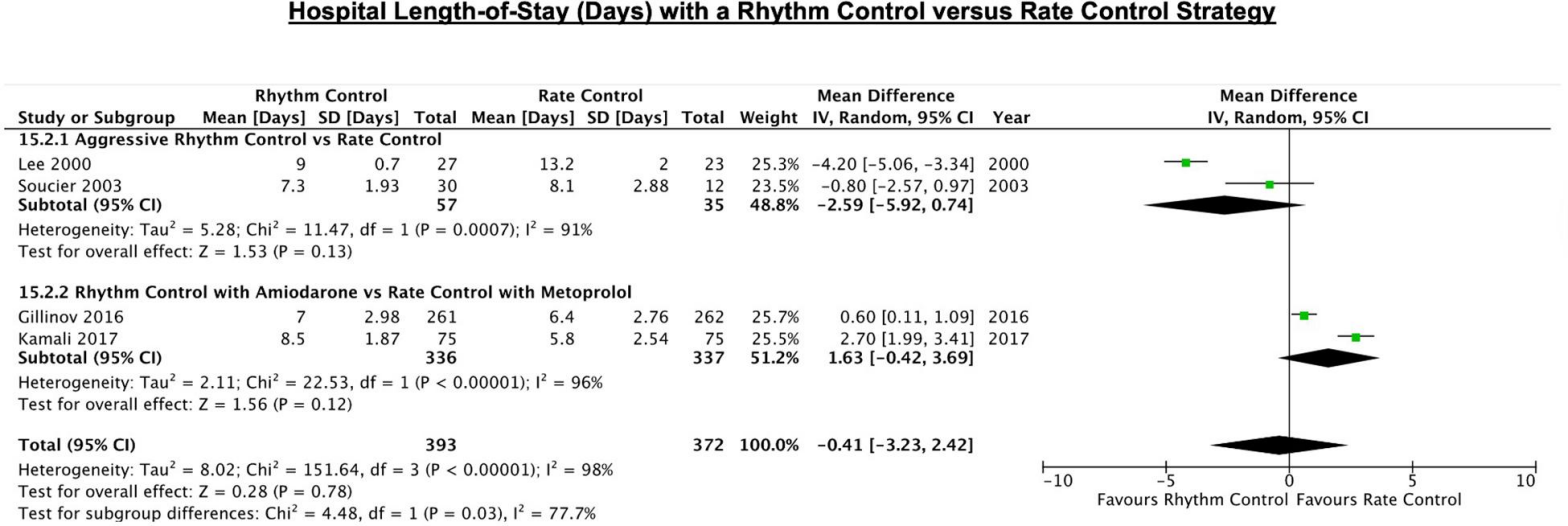
Hospital length of stay (days) with a rhythm versus rate control strategy. Aggressive rhythm control includes early or active rhythm restoration using any of the following: electrical cardioversion, intravenous procainamide, flecainide, sotalol, ibutilide, or propafenone. Rate control strategies include the use of beta-blockers, calcium channel blockers, or digoxin.

A total of 4 RCTs (*n* = 765) evaluated the hospital LOS, and an all-studies-included analysis revealed no difference between rhythm control and rate control interventions [MD: −0.41 days (95% CI: −3.23, 2.42), *p* = 0.78, *I*^2^ = 98%] [Field, (16, 18–20)]. Given the observed heterogeneity, we performed an exploratory subgroup analysis, categorizing rhythm control strategies as “aggressive” vs. “conventional”. This categorization was developed *post hoc* to explore potential sources of statistical heterogeneity. The subgroups were found to be different (test for subgroup differences *p* = 0.03), and the direction of effect size estimates became concordant when studies exploring aggressive rhythm control strategies were analyzed separately from those exploring less aggressive strategies (amiodarone). The effect estimates trended towards a shorter hospital length of stay with aggressive rhythm control, compared to rate control [MD: −2.59 (95% CI: −5.92, 0.74), *p* = 0.13, *I*^2^ = 91%]. Conversely, less aggressive rhythm control with amiodarone exhibited a trend towards longer hospital length of stay compared to rate control [MD: 1.63 (95% CI: −0.42, 3.69), *p* = 0.12, *I*^2^ = 96%]. Despite providing insight into the underlying mechanisms for heterogeneity, these associations did not attain statistical significance (Figure 4).

#### In-hospital conversion to normal sinus rhythm

**Figure 5:**
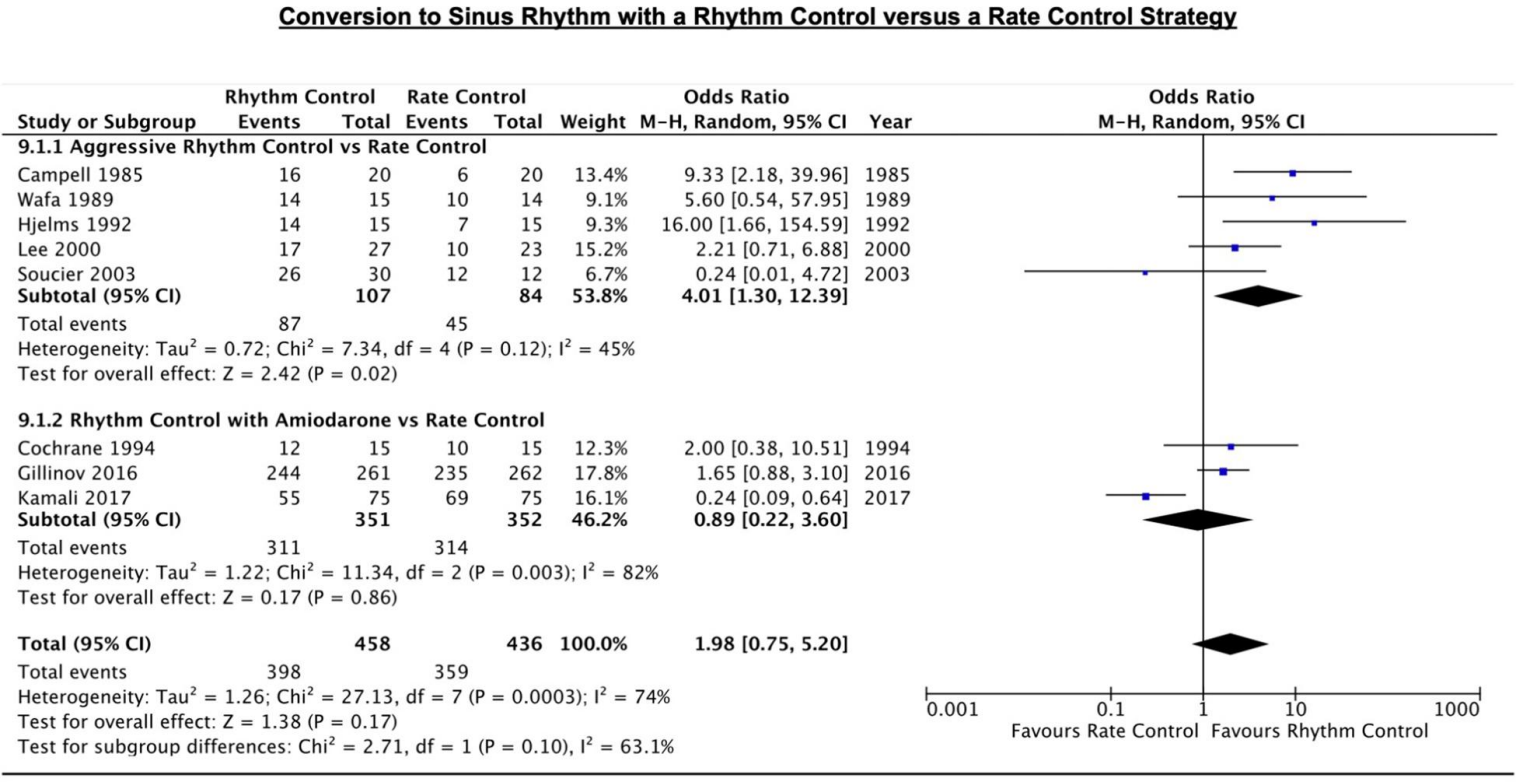
Conversion to normal sinus rhythm (odds ratio) with a rhythm versus rate control strategy. Aggressive rhythm control includes early or active rhythm restoration using any of the following: electrical cardioversion, intravenous procainamide, flecainide, sotalol, ibutilide, or propafenone. Rate control strategies include the use of beta-blockers, calcium channel blockers, or digoxin.

Eight studies (*n* = 894 patients) reported rates of in-hospital conversion to NSR (15–22). The effect estimate for all studies combined revealed no difference between a rhythm control and a rate control strategy [OR for conversion to sinus rhythm: 1.98 (95% CI: 0.75, 5.20), *p* = 0.17, *I*^2^ = 74%]. There was, however, a remarkable variation in the employed rhythm control modalities; while some studies adopted a more aggressive rhythm control approach (e.g., procainamide, propafenone, or electric cardioversion), others used a less aggressive approach with amiodarone. It was therefore prudent to analyze the aggressive rhythm control and amiodarone subgroups again separately. Higher odds of conversion to normal sinus rhythm with aggressive rhythm control strategies were observed compared to rate control [OR: 4.01 (95% CI: 1.30, 12.39), *p* = 0.02, *I*^2^ = 45%]. The odds of conversion to sinus rhythm with amiodarone only were no different from rate control [OR: 0.89 (95% CI: 0.22–3.60, *p* = 0.86, *I*^2^ = 82%)] (Figure 5). The test for subgroup differences, however, revealed no significant difference between the subgroups (*p* = 0.10).

#### Medication adverse effects (hypotension, bradycardia, and syncope)

**Figure 6:**
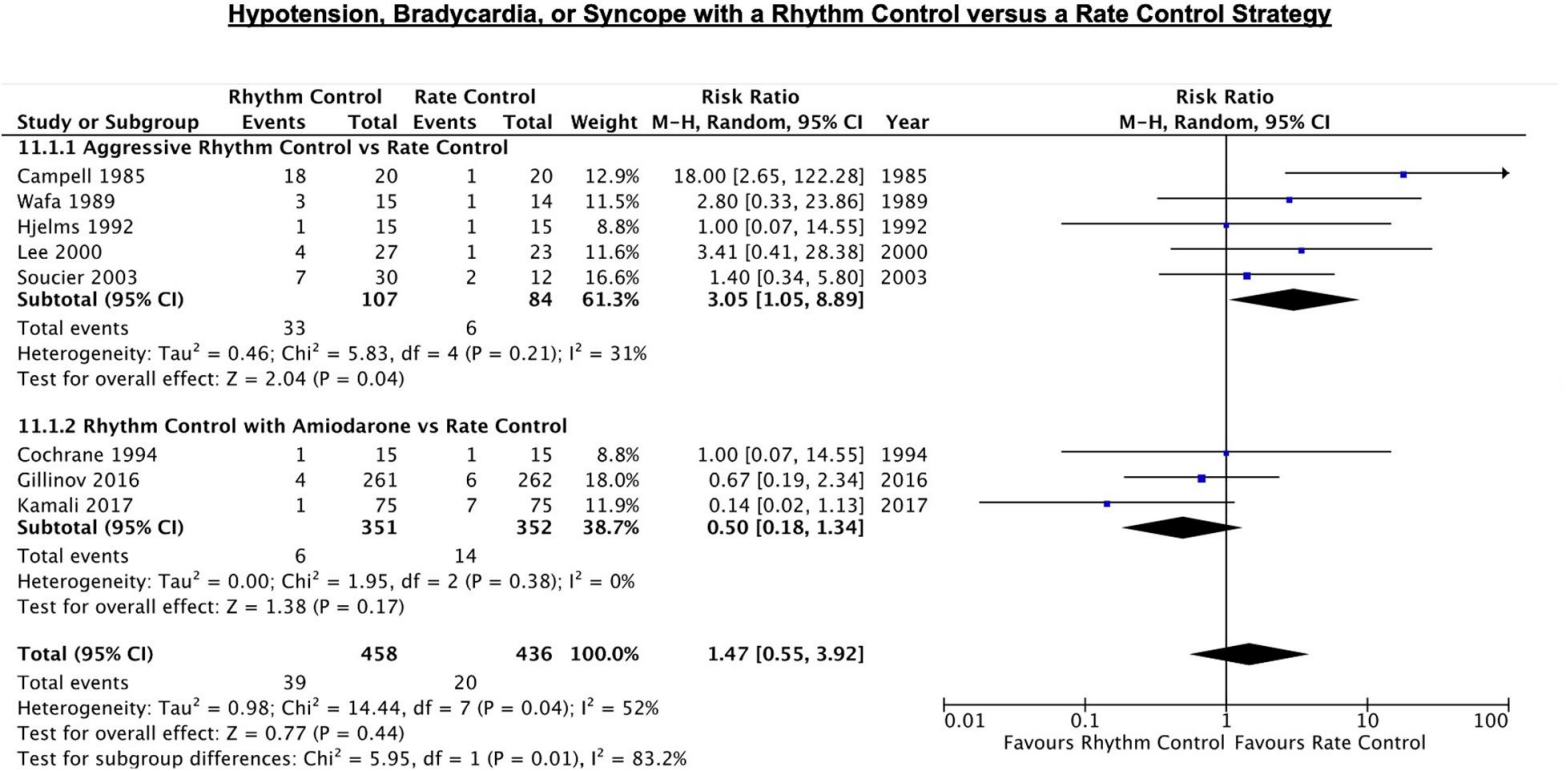
Hypotension, bradycardia, or syncope (risk ratio) with a rhythm versus rate control strategy. Aggressive rhythm control includes early or active rhythm restoration using any of the following: electrical cardioversion, intravenous procainamide, flecainide, sotalol, ibutilide, or propafenone. Rate control strategies include the use of beta-blockers, calcium channel blockers, or digoxin.

All included studies (*n* = 894 patients) reported the incidence of medication-related hemodynamic adverse events (bradycardia, hypotension, and/or syncope). As a composite outcome, these adverse events were comparable between rhythm and rate control strategies [RR: 1.47 (95% CI: 0.55, 3.92), *p* = 0.44, *I*^2^ = 52%] (15–22). However, subgroup analysis revealed a higher proportion of hypotension, bradycardia, or syncope with an aggressive rhythm control strategy (30.8%, SD: 4.4%) compared with a rate control strategy (7.1%, SD: 2.8%). This increased incidence of hypotension, bradycardia, and/or syncope with an aggressive rhythm control strategy was statistically significant [RR: 3.05 (95% CI: 1.05–8.89); *p* = 0.04; *I*^2^ = 31%]. Conversely, amiodarone was not associated with a higher risk of hemodynamic adverse effects compared with rate control [RR: 0.50 (95% CI: 0.18, 1.34), *p* = 0.17, *I*^2^ = 0%] (Figure 6). There was a significant between-subgroup difference in effect sizes (*p* = 0.01).

#### Congestive heart failure/pulmonary edema

Compared with rate control interventions, rhythm control showed no significant difference in the rate of new-onset CHF/pulmonary edema across the three studies (*n* = 615) reporting this outcome [OR: 1.09 (95% CI: 0.46, 2.61, *p* = 0.84, *I*^2^ = 0)] (16, 19, 20).

#### Thromboembolic events

Only two studies had sufficient follow-up to report this outcome (*n* = 565). The pooled effect showed no difference in the rate of thromboembolic events between the two groups [OR: 1.48 (95% CI: 0.31, 7.16), *p* = 0.62, *I*^2^ = 23%] (16, 20). Given the limited number of studies, the pooled effect estimate should be interpreted with caution.

#### Mortality

**Figure 7:**
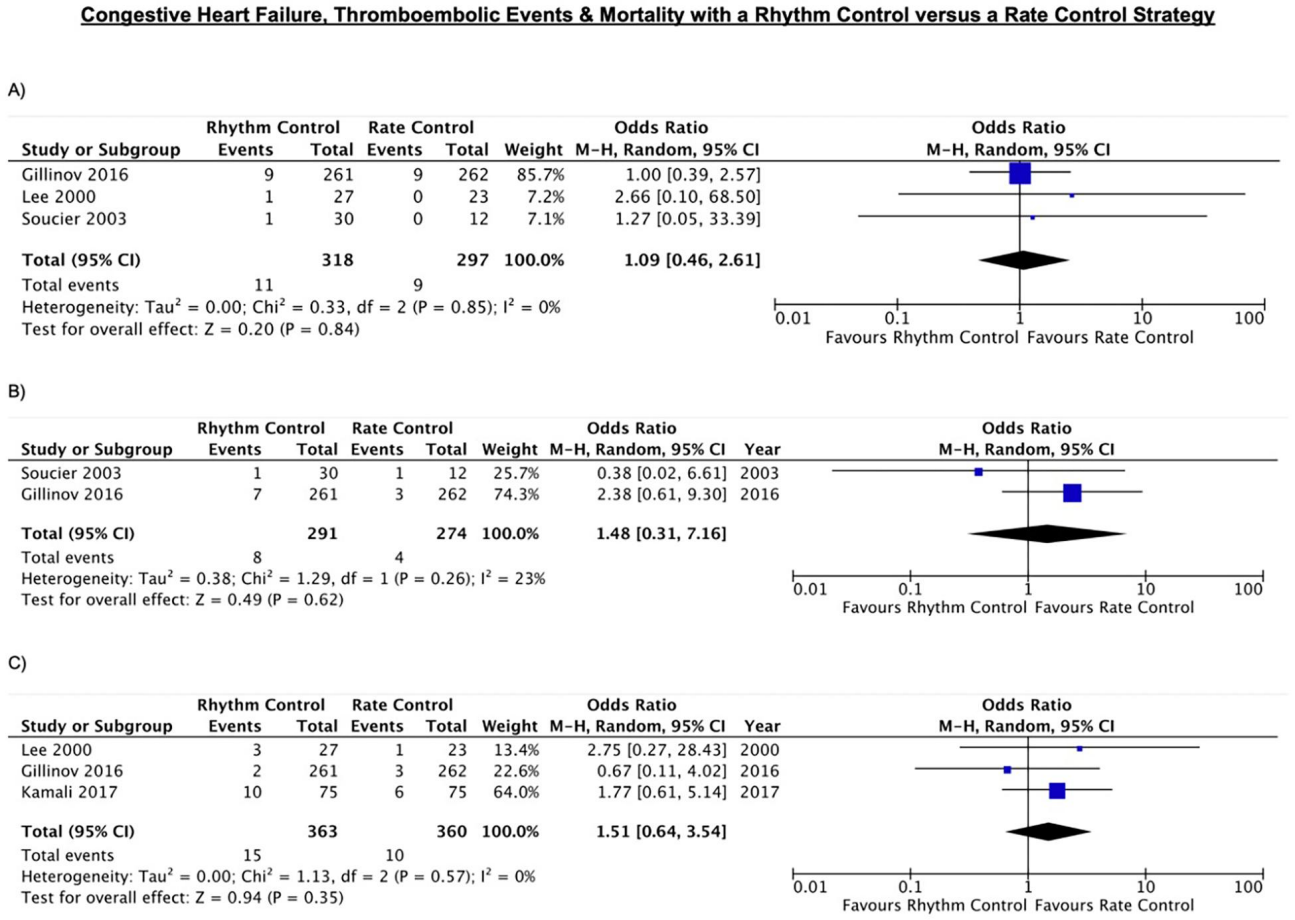
Congestive heart failure/pulmonary edema, thromboembolic events, and mortality (odds ratio) with a rhythm control versus a rate control strategy. **(A)** Congestive heart failure and pulmonary edema with rhythm control versus rate control strategies. **(B)** Thromboembolic events with rhythm control versus rate control strategies. **(C)** Mortality with rhythm control versus rate control strategies.

Only three RCTs reported mortality rates for both strategies in our patient population (*n* = 723). The overall effect was similar; adopting either strategy conferred no difference in mortality [OR: 1.51 (95% CI: 0.64–3.54, *p* = 0.35, *I*^2^ = 0%)] (Figure 7) (16, 18, 19).

#### Assessment of publication bias

**Figure 8:**
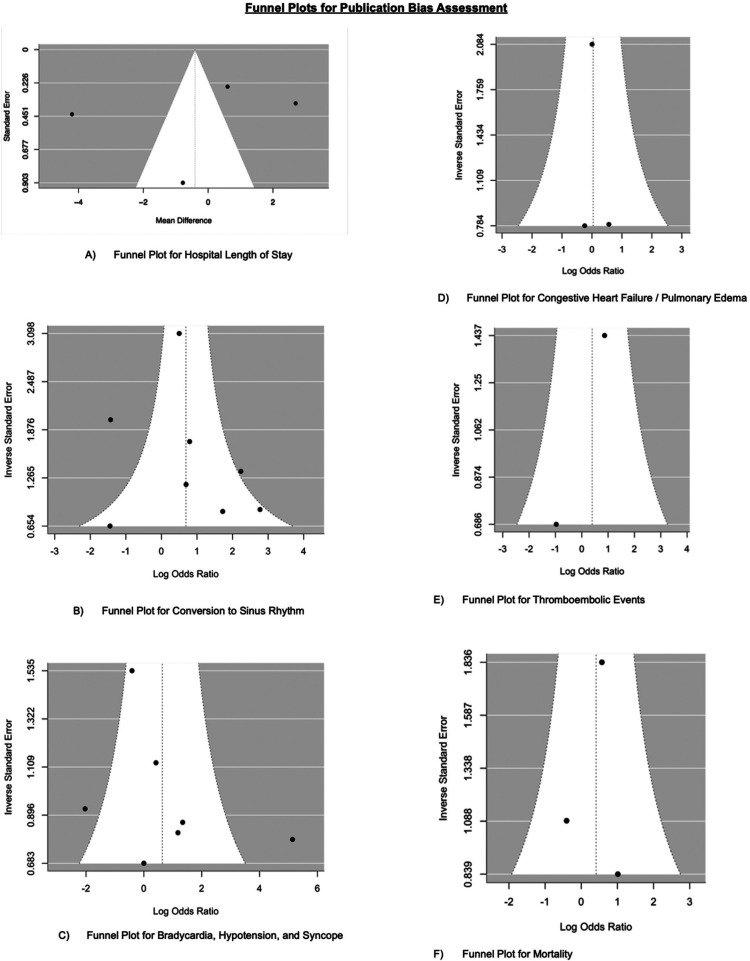
Publication bias assessment for **(A)** hospital length of stag, **(B)** conversion to normal sinus rhythm, **(C)** bradycardia, hypotension, and syncope, **(D)** congestive heart failure/pulmonary edema, **(E)** thromboembolic events, and **(F)** mortality.

Contour-enhanced funnel plots were generated (Figure 8), and Egger's regression test was used to assess funnel plot asymmetry. There was no evidence of funnel plot asymmetry for hospital length of stay (*p* = 0.73), conversion to sinus rhythm (*p* = 0.60), hypotension/bradycardia/syncope (*p* = 0.48), congestive heart failure/pulmonary edema (*p* = 0.88), or mortality (*p* = 0.92). However, given the limited number of included studies, Egger's regression results should be interpreted with caution. Notably, our trial registry search did not identify any registered unpublished trials. This suggests a true paucity of studies examining patients with post-cardiac surgery atrial fibrillation rather than a “file drawer effect”.

#### Strength of evidence using the GRADE criteria

The strength of the evidence was very low regarding the effect of either strategy on hospital length of stay; however, only 4 RCTs met inclusion criteria for this outcome. Conversion to normal sinus rhythm was more likely with a rhythm control strategy, with moderate certainty of evidence. The evidence for medication-related adverse effects (hypotension, bradycardia, or syncope) with a rhythm control strategy was deemed of very low certainty, and this result was robust to sensitivity analysis. The effect of either strategy on the incidence of heart failure, thromboembolic events, and mortality was of low to very low certainty. Details of the GRADE assessment for all study outcomes are listed in Table 3.

**Table 3:** Summary of findings & strength of evidence per the grading of recommendations, assessment, development and evaluations (GRADE) framework.

Summary of findings:						
Rhythm control compared to Rate control for Postoperative Atrial Fibrillation in Cardiac Surgery						
Patient or population: Postoperative Atrial Fibrillation in Cardiac Surgery						
Setting: RCTs						
Intervention: Rhythm control						
Comparison: Rate control						

Outcomes	Anticipated absolute effects* (95% CI)		Relative effect (95% CI)	No of participants (studies)	Certainty of the evidence (GRADE)	Comments
	Risk with Rate control	Risk with Rhythm control				
Hospital length of stay (LOS) assessed with: Days	The mean hospital length of stay was **8.4** days	MD: **0.41 days lower** (3.23 lower to 2.42 higher)	—	765 (4 RCTs)	⊕◯◯◯ VERY LOW^a^^,^^b^^,^^c^	Neither strategy is superior in reducing the LOS. More recent trials indicate a decreasing trend of LOS for rate control trials, perhaps reflective of acceptance of a higher target heart rate.
In-hospital conversion to sinus rhythm (NSR) assessed with: Assessment of heart rhythm	823 per 1,000	**902 per 1,000** (777–960)	**OR**: **1.98** (0.75–5.20)	894 (8 RCTs)	⊕⊕⊕◯ MODERATE^a^	Exclusion of the Kamali study yielded an OR of 3.66 (1.88–7.12). This implied that rhythm control therapy had a higher success rate at converting patients with POAF into sinus rhythm.
Thromboembolic events (TE) assessed with Stroke, pulmonary or venous embolism	15 per 1,000	**22 per 1,000** (5–98)	**OR**: **1.48** (0.31–7.16)	565 (2 RCTs)	⊕⊕◯◯ LOW^a^^,^^c^	No significant difference in the rate of thromboembolic events with either strategy. Rhythm control may present an effective option when anticoagulant use is associated with a higher risk of bleeding.
Heart failure (HF) assessed with: New CHF or pulmonary edema	37 per 1,000	**40 per 1,000** (17–91)	**OR**: **1.09** (0.46–2.61)	615 (3 RCTs)	⊕⊕◯◯ LOW^a^^,^^c^	No significant differences in the rate of heart failure with either strategy.
Drug adverse effects (AE) assessed with: Hypotension, bradycardia & syncope	46 per 1,000	**68 per 1,000** (25–180)	**OR**: **1.47** (0.55–3.92)	894 (8 RCTs)	⊕◯◯◯ VERY LOW^a^^,^^c^	No significant differences in drug-related adverse effects with either strategy. This result was robust to sensitivity analysis.
Mortality (Deaths) assessed with the number of deaths	28 per 1,000	**42 per 1,000** (18–93)	**OR**: **1.51** (0.64–3.54)	723 (3 RCTs)	⊕◯◯◯ VERY LOW^a^^,^^c^	As with studies in noncardiac surgery, rhythm control does not afford mortality benefit in cardiac surgery.

* **The risk in the intervention group** (and its 95% confidence interval) is based on the assumed risk in the comparison group and the **relative effect** of the intervention (and its 95% CI). CI, confidence interval; MD, mean difference; OR, odds ratio.

**GRADE Working Group grades of evidence.**

**High certainty:** We are very confident that the true effect lies close to that of the estimate of the effect.

**Moderate certainty:** We are moderately confident in the effect estimate: The true effect is likely to be close to the estimate of the effect, but there is a possibility that it is substantially different.

**Low certainty:** Our confidence in the effect estimate is limited: The true effect may be substantially different from the estimate of the effect.

**Very low certainty:** We have very little confidence in the effect estimate: The true effect is likely to be substantially different from the estimate of effect.

^a^ downgraded for risk of bias.

^b^ downgraded for inconsistency (unexplained heterogeneity).

^c^ downgraded for imprecision.

## Discussion

Our systematic review and meta-analysis found no significant difference in hospital LOS between rhythm and rate control strategies. Aggressive rhythm control interventions (ibutilide, procainamide, propafenone, and electric cardioversion) were more likely to achieve in-hospital conversion to sinus rhythm than rate control, but they were also associated with a higher risk of medication-related side effects. Amiodarone in-hospital conversion rates were no different from rate control. Given the limited power and insufficient follow-up periods, the impact of either treatment strategy on long-term outcomes remains unclear.

Despite higher conversion rates to sinus rhythm with aggressive rhythm control, this did not translate into shorter hospital stays in the two studies that examined this outcome. The higher rates of medication-related hypotension, bradycardia, and syncope with aggressive rhythm control are biologically plausible, as agents such as procainamide and propafenone are known to have strong negative inotropic effects (23). Our results therefore support close monitoring and possibly pre-emptive intervention when such aggressive rhythm control agents are used in the cardiac surgery population.

There was no evidence that amiodarone was superior to rate control in achieving normal sinus rhythm in postcardiac surgery patients. In addition, the use of amiodarone was not associated with a shorter hospital LOS compared to rate control; rather, there was a non-significant trend towards a longer hospital stay. Amiodarone has a much slower onset than other antiarrhythmics, which may render it an unfavorable choice for an often self-terminating arrhythmia (24, 25). The incidence of medication-related adverse events (composite of hypotension, bradycardia, and syncope) with amiodarone in this meta-analysis was no different from rate control. This was not surprising, as amiodarone has long been known to be a hemodynamically well-tolerated agent compared to other antiarrhythmics (26).

Thromboembolic events were reported in only 2 studies, with both treatment strategies (rate vs. rhythm control) appearing comparable (16, 20). Given that rate control is almost always combined with anticoagulation, however, this lack of difference would be expected. Similarly, our review did not find a mortality benefit of any treatment strategy over the other. These results are consistent with other meta-analyses in the noncardiac surgery population (27). Despite the pathophysiological triggers and underlying mechanisms of POAF in cardiac surgery patients being very distinct from the general atrial fibrillation (AF) population, it is quite possible that either management strategy would still carry no impact on mortality. In addition, we did not find any difference between the two strategies regarding the development of congestive heart failure or pulmonary edema as a complication of treatment and/or the AF (17, 20).

Our qualitative literature review supports an individualized patient approach to POAF treatment. Patients with hemodynamic instability clearly attributable to new-onset POAF may warrant synchronized electrical cardioversion; however, a higher recurrence rate remains problematic if the underlying drivers are not corrected concomitantly (28). It is therefore important to expeditiously address any underlying drivers of atrial fibrillation and to consider antiarrhythmic therapy before cardioversion to achieve a more sustained effect. In patients with POAF who are hemodynamically stable, our review suggests that either a rate-control strategy or the use of amiodarone is a reasonable option until spontaneous reversion to sinus rhythm occurs. If spontaneous reversion does not occur within 48–72 h, anticoagulation should be initiated in the absence of contraindications (29). Patients with relative or absolute contraindications to anticoagulation warrant an initial pharmacologic rhythm-control approach. If refractory, electrical cardioversion at the 48-hour mark (before left atrial clot formation) may be reasonable. Given the high incidence of recurrence, continued pharmacologic therapy post-cardioversion may be considered (30).

Preventive strategies in the preoperative setting may also help reduce the incidence of POAF. These include optimization of electrolyte imbalances, continuation or initiation of beta-blockers where appropriate, and the use of prophylactic antiarrhythmic agents in high-risk patients. In addition, addressing modifiable risk factors such as volume status and systemic inflammation may further reduce the likelihood of POAF development. Future studies should explore the integration of such preventive strategies with postoperative management approaches (7, 31).

Although there was no evidence that either treatment strategy was superior, this meta-analysis calls for further research with well-designed randomized trials focused on clinically meaningful outcomes (hospital length of stay, rates of thromboembolic events, and mortality). Any further research in this domain must include longer follow-up periods. In addition, more aggressive rhythm-control strategies (such as propafenone, ibutilide, and cardioversion) showed some promise in terms of conversion efficacy and warrant further investigation.

Our study has several strengths. It is the first systematic review and meta-analysis to examine the short- and long-term effects of either treatment strategy for new-onset POAF. We employed a comprehensive search strategy across multiple databases. We included only data from RCTs. We followed a robust methodology, used subgroup analyses to assess heterogeneity where appropriate, and graded the strength of our findings. Finally, our exploratory evaluation of secondary outcomes was hypothesis-generating. Our analysis aimed to avoid overstating or inflating any possible type I error. These factors emphasize the validity of our results.

However, our current work has a few limitations. First, our search retrieved a small number of RCTs (*k* = 8), highlighting the paucity of clinical trials in this domain. Moreover, only two RCTs had sample sizes exceeding 100 patients (16, 18). Second, the classification of “aggressive” vs. “conventional” rhythm control strategies was developed *post hoc* to assess potential sources of heterogeneity and should therefore be viewed as exploratory. This approach was necessitated by considerable clinical heterogeneity in treatment approaches among the included studies. The lack of patient-level data precluded the use of more powerful analytic techniques. Additionally, effect estimates for some outcomes (e.g., thromboembolic events) were derived from a very limited number of studies. Producing robust effect estimates and exploring heterogeneity under such constraints may not be feasible. Similarly, variability in follow-up duration across trials in our meta-analysis may limit the generalizability of important outcomes such as mortality. Furthermore, several subgroup analyses were based on a small number of studies, thereby reducing statistical power and limiting the reliability of subgroup-specific conclusions. Subgroup analysis findings should therefore be considered hypothesis-generating.

## Conclusion

This meta-analysis found no evidence that a rhythm-control strategy reduced hospital LOS compared with a rate-control strategy in postcardiac-surgery patients. Although an aggressive rhythm-control strategy achieved higher rates of conversion to sinus rhythm, it was associated with a higher risk of medication-related hypotension and did not translate into fewer complications (thromboembolic events, heart failure, and mortality). Consequently, rate control with anticoagulation appears to be as effective as rhythm control for managing POAF after cardiac surgery.

## Data Availability

The original contributions presented in the study are included in the article/Supplementary Material, further inquiries can be directed to the corresponding author.
