# Supplementary material for: Rhythm vs. rate control for treatment of postoperative atrial fibrillation after cardiac surgery: a systematic review and meta-analysis of randomized controlled trials
Source: Front Cardiovasc Med. 2026 Jul 14;13:1820175. doi: 10.3389/fcvm.2026.1820175 (PMC13407837; doi:10.3389/fcvm.2026.1820175)
Supplement: Supplementary file 1 [file Datasheet1.pdf]

Rhythm versus Rate Control for Treatment of Postoperative Atrial Fibrillation after Cardiac Surgery: A Systematic Review and Meta-analysis of Randomized Controlled Trials

Mohammad Dairi, Mohammed Tarabzoni, Christopher Tarola, Herman Sehmbi, Hassan Alwafi, Saeed M. Mardy Alghamdi, Sariya Khan, Waleed T. Alotaibi, Ahmed F. Hegazy

SUPPLEMENTARY APPENDIX

Supplementary Appendix 1:

Outcome Definitions

Outcome	Definition	Measurement Unit
Hospital length of stay	Postoperative or postrandomization hospital length of stay for the index cardiac surgery admission	Days
In-hospital conversion to normal sinus rhythm	Number of patients converted to normal sinus rhythm at latest reported time-point during index cardiac surgery admission	Frequency
Medication adverse effects	Number of patients developing any of the following: hypotension, bradycardia, or syncope during study follow-up	Frequency
Incidence of congestive heart failure & pulmonary edema	Number of patients developing new-onset heart failure or pulmonary edema during study follow-up	Frequency
Thromboembolic events	Number of patients developing any thromboembolic event (cerebrovascular or non-cerebrovascular) during study follow-up	Frequency
Mortality	Number of postcardiac surgery deaths during study follow-up	Frequency

Supplementary Appendix 2:

Literature Search

Date: October 19, 2025

Databases Used:

- Medline (ALL)
- PubMed-NOT-Medline (NLM)
- Embase
- Cochrane Central Register of Controlled Trials
- CINAHL
- Web of Science
- Scopus
- ProQuest Dissertations
- ClinicalTrials.gov

Notes:

- Final searches were run on October 17-18, 2025
- www.accessdata.fda.gov/scripts/cder/drugsatfda/ search was not included, as it is only a database of drugs

Results by Database:

**Database**	**Results**
Medline & Medline in-process	327
PubMed-NOT-Medline (NLM)	14
Embase	1448
Cochrane Central Register of Controlled Trials (CENTRAL)	106
CINAHL	69
Web of Science	370
Scopus	337
ProQuest Dissertations & Theses Global	0
Duplicates Removed	*911*
**Total**	**1760**
ClinicalTrials.gov (not included in total above.)	413

Search Strategies:

<u>Medline</u>

Database(s): **Ovid MEDLINE(R) ALL** 1946 to October 16, 2025
Search Strategy:

#	Searches	Results
1	Atrial fibrillation.af.	71520
2	Atrial Fibrillation/	47859
3	or/1-2	71520
4	(cardiac surger* or open?heart surger*).af.	63407
5	exp Cardiac Surgical Procedures/	201602
6	or/4-5	233588
7	3 and 6	6733
8	(Rhythm control or anti-arrhythmi* or electric countershock or amiodarone or procainamide or propafenone or flecainide or disopyramide or quinidine or ibutilide or cardioversion or sotalol).af.	61112
9	(Anti-Arrhythmia Agents or Amiodarone or Procainamide or Propafenone or Sotalol).sh.	34274
10	exp Electric Stimulation Therapy/	72742
11	or/8-10	117906
12	(Rate control or beta-block* or metoprolol or bisoprolol or beta-Adrenergic Receptor Block* or beta-Adrenergic Blocking Agent* or Calcium Channel Blocker* or Calcium Channel Antagonist* or Calcium Channel Blocking Drug* or exogenous Calcium Inhibitor* or Exogenous Calcium Antagonist* or Exogenous Calcium Blockader* or verapamil or diltiazem or digoxin* or Lanacordin or Lanicor or Lanoxicaps or Dilanacin or Digacin or Digitek or Digoregen or Mapluxin or Lanoxin or Lenoxin or propranolol or Inderal or esmolol or brevibloc).af.	155011
13	(Calcium Channel Blockers or Digoxin).sh.	46528
14	exp Adrenergic beta-Antagonists/	81383
15	or/12-14	179810
16	7 and 11 and 15	327

PubMed (not Medline)

((Atrial fibrillation[all] OR Atrial Fibrillation[mesh]) AND ((cardiac surger*[all] OR open heart surger*[all] OR open-heart surger*[all]) OR Cardiac Surgical Procedures[mesh])
AND
(Rhythm control[all] OR anti-arrhythmi*[all] OR electric countershock[all] OR amiodarone[all] OR procainamide[all] OR propafenone[all] OR flecainide[all] OR disopyramide[all] OR quinidine[all] OR ibutilide[all] OR cardioversion[all] OR sotalol[all] OR Anti-Arrhythmia Agents[mesh] OR Amiodarone[mesh] OR Procainamide[mesh] OR Propafenone[mesh] OR Sotalol[mesh] OR Electric Stimulation Therapy[mesh])
AND
(Rate control[all] OR beta-block*[all] OR metoprolol[all] OR bisoprolol[all] OR beta-Adrenergic Receptor Block[all] OR beta-Adrenergic Blocking Agent*[all] OR Calcium Channel Blocker*[all] OR Calcium Channel Antagonist*[all] OR Calcium Channel Blocking Drug*[all] OR exogenous Calcium Inhibitor*[all] OR Exogenous Calcium Antagonist*[all] OR Exogenous Calcium Blockader*[all] OR verapamil[all] OR diltiazem[all] OR digoxin*[all] OR Lanacordin[all] OR Lanicor[all] OR Lanoxicaps[all] OR Dilanacin[all] OR Digacin[all] OR Digitek[all] OR Digoregen[all] OR Mapluxin[all] OR Lanoxin[all] OR Lenoxin[all] OR propranolol[all] OR Inderal[all] OR esmolol[all] OR brevibloc[all] OR Adrenergic beta-Antagonists[mesh] OR Calcium Channel Blockers[mesh] OR Digoxin[mesh])

AND (((((publisher[sb] NOT pubstatusnihms NOT pubstatuspmcsd NOT pmcbook) OR inprocess[sb] OR pubmednotmedline[sb] OR ((pubstatusnihms OR pubstatuspmcsd) AND publisher[sb])))))

Embase

Database(s): **Embase Classic+Embase** 1947 to 2025 October 16
Search Strategy:

#	Searches	Results
1	Atrial fibrillation.af.	120120
2	exp Atrial Fibrillation/	45866
3	or/1-2	120120
4	(cardiac surger* or open?heart surger*).af.	108315
5	exp heart surgery/	345621
6	or/4-5	378631
7	3 and 6	16641
8	(Rhythm control or anti-arrhythmi* or electric countershock or amiodarone or procainamide or propafenone or flecainide or disopyramide or quinidine or ibutilide or cardioversion or sotalol).af.	97330
9	exp Anti-Arrhythmia Agent/	341059
10	(Amiodarone or Procainamide or Propafenone or Sotalol).sh.	54325
11	exp electrotherapy/	225358
12	or/8-11	555332
13	(Rate control or beta-block* or metoprolol or bisoprolol or beta-Adrenergic Receptor Block* or beta-Adrenergic Blocking Agent* or Calcium Channel Blocker* or Calcium Channel Antagonist* or Calcium Channel Blocking Drug* or exogenous Calcium Inhibitor* or Exogenous Calcium Antagonist* or Exogenous Calcium Blockader* or verapamil or diltiazem or digoxin* or Lanacordin or Lanicor or Lanoxicaps or Dilanacin or Digacin or Digitek or Digoregen or Mapluxin or Lanoxin or Lenoxin or propranolol or Inderal or esmolol or brevibloc).af.	346498
14	exp beta adrenergic receptor blocking agent/	287399
15	exp calcium channel blocking agent/	212917
16	Digoxin/	44776
17	or/13-16	484230
18	7 and 12 and 17	1448

Cochrane Central Register of Controlled Trials

Database(s): **EBM Reviews - Cochrane Central Register of Controlled Trials** September 2025
Search Strategy:

#	Searches	Results
1	Atrial fibrillation.af.	9138
2	Atrial Fibrillation/	3757
3	or/1-2	9138
4	(cardiac surger* or open?heart surger*).af.	6798
5	exp Cardiac Surgical Procedures/	12107
6	or/4-5	16263
7	3 and 6	801
8	(Rhythm control or anti-arrhythmi* or electric countershock or amiodarone or procainamide or propafenone or flecainide or disopyramide or quinidine or ibutilide or cardioversion or sotalol).af.	5221
9	(Anti-Arrhythmia Agents or Amiodarone or Procainamide or Propafenone or Sotalol).sh.	2214
10	exp Electric Stimulation Therapy/	5660
11	or/8-10	9932
12	(Rate control or beta-block* or metoprolol or bisoprolol or beta-Adrenergic Receptor Block* or beta-Adrenergic Blocking Agent* or Calcium Channel Blocker* or Calcium Channel Antagonist* or Calcium Channel Blocking Drug* or exogenous Calcium Inhibitor* or Exogenous Calcium Antagonist* or Exogenous Calcium Blockader* or verapamil or diltiazem or digoxin* or Lanacordin or Lanicor or Lanoxicaps or Dilanacin or Digacin or Digitek or Digoregen or Mapluxin or Lanoxin or Lenoxin or propranolol or Inderal or esmolol or brevibloc).af.	23037
13	(Calcium Channel Blockers or Digoxin).sh.	3470
14	exp Adrenergic beta-Antagonists/	10330
15	or/12-14	26381
16	7 and 11 and 15	106

CINAHL

S14	S7 AND S10 AND S13	69
S13	S11 OR S12	18,192
S12	(MH "Adrenergic Beta-Antagonists+") OR (MH "Calcium Channel Blockers+") OR (MH "Digoxin")	10,280
S11	TI(Rate control or beta-block* or metoprolol or bisoprolol or beta-Adrenergic Receptor Block* or beta-Adrenergic Blocking Agent* or Calcium Channel Blocker* or Calcium Channel Antagonist* or Calcium Channel Blocking Drug* or exogenous Calcium Inhibitor* or Exogenous Calcium Antagonist* or Exogenous Calcium Blockader* or verapamil or diltiazem or digoxin* or Lanacordin or Lanicor or Lanoxicaps or Dilanacin or Digacin or Digitek or Digoregen or Mapluxin or Lanoxin or Lenoxin or propranolol or Inderal or esmolol or brevibloc) OR AB(Rate control or beta-block* or metoprolol or bisoprolol or beta-Adrenergic Receptor Block* or beta-Adrenergic Blocking Agent* or Calcium Channel Blocker* or Calcium Channel Antagonist* or Calcium Channel Blocking Drug* or exogenous Calcium Inhibitor* or Exogenous Calcium Antagonist* or Exogenous Calcium Blockader* or verapamil or diltiazem or digoxin* or Lanacordin or Lanicor or Lanoxicaps or Dilanacin or Digacin or Digitek or Digoregen or Mapluxin or Lanoxin or Lenoxin or propranolol or Inderal or esmolol or brevibloc)	12,078
S10	S8 OR S9	23,518
S9	(MH "Antiarrhythmia Agents+") OR (MH "Amiodarone+") OR (MH "Procainamide") OR (MH "Propafenone") OR (MH "Sotalol") OR (MH "Electrotherapy+")	21,790
S8	TI(Rhythm control or anti-arrhythmi* or electric countershock or amiodarone or procainamide or propafenone or flecainide or disopyramide or quinidine or ibutilide or cardioversion or sotalol) OR AB(Rhythm control or anti-arrhythmi* or electric countershock or amiodarone or procainamide or propafenone or flecainide or disopyramide or quinidine or ibutilide or cardioversion or sotalol)	3,280
S7	S3 AND S6	1,018

S6	S4 OR S5	28,717
S5	(MH "Heart Surgery+")	26,288
S4	TI(cardiac surger* OR open?heart surger*) OR AB(cardiac surger* OR open?heart surger*)	6,061
S3	S1 OR S2	15,824
S2	(MH "Atrial Fibrillation")	11,786
S1	TI(Atrial fibrillation) OR AB(Atrial fibrillation)	12,738

Web of Science

# 4	**370**	#3 AND #2 AND #1<br>*Indexes=SCI-EXPANDED, SSCI, A&HCI, CPCI-S, CPCI-SSH, ESCI Timespan=All years*
# 3	**943,224**	TS=(Rate control or beta-block* or metoprolol or bisoprolol or beta-Adrenergic Receptor Block* or beta-Adrenergic Blocking Agent* or Calcium Channel Blocker* or Calcium Channel Antagonist* or Calcium Channel Blocking Drug* or exogenous Calcium Inhibitor* or Exogenous Calcium Antagonist* or Exogenous Calcium Blockader* or verapamil or diltiazem or digoxin* or Lanacordin or Lanicor or Lanoxicaps or Dilanacin or Digacin or Digitek or Digoregen or Mapluxin or Lanoxin or Lenoxin or propranolol or Inderal or esmolol or brevibloc)<br>*Indexes=SCI-EXPANDED, SSCI, A&HCI, CPCI-S, CPCI-SSH, ESCI Timespan=All years*
# 2	**62,202**	TS=(Rhythm control or anti-arrhythmi* or electric countershock or amiodarone or procainamide or propafenone or flecainide or disopyramide or quinidine or ibutilide or cardioversion or sotalol or electric stimulation therapy)<br>*Indexes=SCI-EXPANDED, SSCI, A&HCI, CPCI-S, CPCI-SSH, ESCI Timespan=All years*
# 1	**4,969**	TS=(Atrial fibrillation) AND TS=(cardiac surger* OR open?heart surger*)<br>*Indexes=SCI-EXPANDED, SSCI, A&HCI, CPCI-S, CPCI-SSH, ESCI Timespan=All years*

Scopus

#1 ((TITLE-ABS-KEY ("Atrial fibrillation")) AND (TITLE-ABS-KEY ("cardiac surger*") OR TITLE-ABS-KEY ("open?heart surger*")))
2,748 documents

#2 (TITLE-ABS-KEY ("Rhythm control" OR anti-arrhythmi* OR "electric countershock" OR amiodarone OR procainamide OR propafenone OR flecainide OR diso pyramide OR quinidine OR ibutilide OR cardioversion OR sotalol OR "electric stimulation therapy"))
120,966 documents

#3 (TITLE-ABS-KEY ("Rate control" OR "beta-block*" OR metoprolol OR bisoprolol OR "beta-Adrenergic Receptor Block*" OR "beta-Adrenergic Blocking Agent*" OR "Calcium Channel Blocker*" OR "Calcium Channel Antagonist*" OR "Calcium Channel Blocking Drug*" OR "exogenous Calcium Inhibitor*" OR "Exogenous Calcium Antagonist*" OR "Exogenous Calcium Blockader*" OR verapamil OR diltiazem OR digoxin* OR lanacordin OR lanicor OR lano xicaps OR dilanacin OR digacin OR digitek OR digoregen OR mapluxin OR lanoxin OR lenoxin OR propranolol OR inderal OR esmolol OR brevibloc))
351,227 documents

#1 AND #2 AND #3
337 documents

ProQuest Dissertations & Theses Global

(noft(Atrial fibrillation) AND noft("cardiac surger*" OR "open?heart surger*")) AND noft("Rhythm control" OR anti-arrhythmi* OR "electric countershock" OR amiodarone OR procainamide OR propafenone OR flecainide OR disopyramide OR quinidine OR ibutilide OR cardioversion OR sotalol OR "electric stimulation therapy") AND noft("Rate control" OR "beta-block*" OR metoprolol OR bisoprolol OR "beta-Adrenergic Receptor Block*" OR "beta-Adrenergic Blocking Agent*" OR "Calcium Channel Blocker*" OR "Calcium Channel Antagonist*" OR "Calcium Channel Blocking Drug*" OR "exogenous Calcium Inhibitor*" OR "Exogenous Calcium Antagonist*" OR "Exogenous Calcium Blockader*" OR verapamil OR diltiazem OR digoxin* OR Lanacordin OR Lanicor OR Lanoxicaps OR Dilanacin OR Digacin OR Digitek OR Digoregen OR Mapluxin OR Lanoxin OR Lenoxin OR propranolol OR Inderal OR esmolol OR brevibloc)
0

noft("Rate control" or "beta-block*" or metoprolol or bisoprolol or "beta-Adrenergic Receptor Block*" or "beta-Adrenergic Blocking Agent*" or "Calcium Channel Blocker*" or "Calcium Channel Antagonist*" or "Calcium Channel Blocking Drug*" or "exogenous Calcium Inhibitor*" or "Exogenous Calcium Antagonist*" or "Exogenous Calcium Blockader*" or verapamil or diltiazem or digoxin* or Lanacordin or Lanicor or Lanoxicaps or Dilanacin or Digacin or Digitek or Digoregen or Mapluxin or Lanoxin or Lenoxin or propranolol or Inderal or esmolol or brevibloc)
4,573

noft("Rhythm control" or anti-arrhythmi* or "electric countershock" or amiodarone or procainamide or propafenone or flecainide or disopyramide or quinidine or ibutilide or cardioversion or sotalol or "electric stimulation therapy")
821

noft(Atrial fibrillation) AND noft("cardiac surger*" OR "open?heart surger*")
29

ClinicalTrials.gov

rhythm control | Atrial Fibrillation

rate control | Atrial Fibrillation
